# Making sense of technology adoption in healthcare: meso-level considerations

**DOI:** 10.1186/s12916-015-0305-8

**Published:** 2015-04-23

**Authors:** Carl R May

**Affiliations:** Faculty of Health Sciences, University of Southampton, Building 67 (Nightingale), University Road, Southampton, SO17 1 BJ UK

**Keywords:** Burden of treatment, Technology adoption, Telecare

## Abstract

It has been clear for some time that the development of telecare faces significant problems. Large scale studies and clinical trials seem to suggest that the cost and clinical effectiveness of telecare systems is doubtful, and the claim that these systems empower or enable service users often seems greatly overstated. The question that stems from this is, can these problems be overcome? Greenhalgh et al. have critiqued the construction of telecare as a generalised technological solution to problems of the delivery of care and have offered a new framework for defining quality in telecare and telehealth. They outline a set of principles that focus on user-centredness, co-creation, integration, and evaluation. This is a valuable approach, and is part of a much wider transformation of the way in which policy and practice researchers conceptualise healthcare delivery as a problem of performativity. Recognising that this is an important shift, in this paper I argue that we also need to keep in mind the meso-level factors that structure new technology applications in practice.

Please see the related article: http://dx.doi.org/10.1186/s12916-015-0279-6

## Background

It has been clear for some time that the development of telecare faces significant problems. Large scale studies and clinical trials seem to suggest that the cost and clinical effectiveness of telecare systems is doubtful [1], and the claim that these systems empower or enable service users often seems greatly overstated [2]. The question that stems from this is, can these problems be overcome? Greenhalgh et al. [3] have critiqued the construction of telecare as a generalised technological solution to problems of the delivery of care, and have offered a new framework for defining quality in telecare and telehealth. They outline a set of principles that focus on the premise that quality telehealth or telecare is i) anchored in a shared understanding of what matters to the user; ii) realistic about the natural history of illness; iii) co-creative, evolving and adapting solutions with users; iv) human, supported through interpersonal relationships and social networks; v) integrated, through attention to mutual awareness and knowledge sharing; and vi) evaluated to drive system learning.

The ARCHIE model outlined by Greenhalgh et al. [3] is useful because it promotes a solution to some of the core problems of adopting, implementing, and normalizing new technologies. In this context, it is part of a much wider shift in the way that health technologies are understood. Since the 1990s, there has been a longstanding movement to reconceptualise health technologies as elements of care that can be co-designed and co-produced [4], and an increasing orientation to theoretical approaches to the investigation and evaluation of new technologies that focuses on human agency and problems of performativity – the things that users do as they work with and around them [5]. The ARCHIE model reflects that shift, while at the same time setting out principles for intervention design that extend far beyond telecare, for it also provides an ethical and a practical framework for thinking about user-centred design across the domain of health and social care research. Indeed, ARCHIE is likely to become rapidly normalised as a structure for thinking about a whole range of complex interventions. However, in thinking about user-centred design and development, it is also worth thinking about where the analysis of Greenhalgh et al. [3], and their new model of practice, stop.

## Discussion

The central focus of the ARCHIE framework is on incorporating users into design processes and then working co-creatively with them in an environment in which interpersonal relations and relational integration are important factors in service development. As a result, analytic attention is directed at local and performative micro-level practices and relations [6,7] as a basis for user-centred design. Highly focused ethnographic and other qualitative studies have played a central role in identifying problems in ‘adoption’, ‘diffusion’, and ‘implementation’ amongst specific groups of users as they engage with new technologies and practices [8]. The results of micro-level ethnographies that focus on performativity are important, but the question is, are they enough?

The argument that I want to make here is that focusing on users increasingly calls on researchers to go further in developing an empirically grounded understanding of the conditions in which technologies like telecare are operationalised and implemented. We need to consider how using ‘new technology’ to mediate between the needs of patients and their families may not compensate for the withdrawal of human relations in healthcare delivery [2], and how shifting burdens of workload and capacity in ‘technological’ care may mean the redistribution of work amongst patients, families, and wider social networks [9], possibly ending with them carrying burdens that they are not strong enough to bear. Meaningful social relations between people who use telecare services and those who provide them are threatened with fragmentation and collapse in such contexts [10]. There is now abundant evidence, especially from Mort and colleagues in the EFORTT Study Group’s work on the practices of telecare, that the rationalisation of care providers’ work practices also negatively affects the call centre operatives who deliver it [11-13].

**Figure 1:**
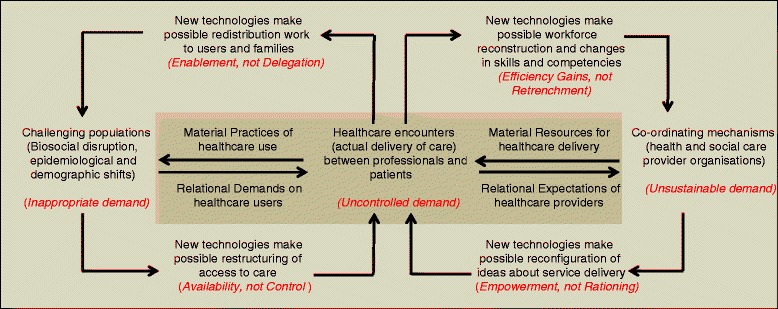
**Focusing on the performative zone does not include the political assumptions that give ‘technology’ structure and meaning.**

Focusing on what happens in the performative domain, as the ARCHIE model does, offers a structure for thinking critically about the underlying technocratic optimism of telecare providers and equipment manufacturers [14]. However, going further in the interests of users might also mean that we have to consider some of the meso-level factors that frame and constrain users in practice. Telecare systems do so much more than provide care. They form a vehicle by which assumptions about demands and eligibility for care, workforce structure, and organisation, shifting burdens of treatment and care, can be articulated and embedded in practice. Figure 1 shows how these assumptions are organised.

Underpinning the demand for telecare has been the notion that the growing numbers of older people with long-term conditions are a challenging population because they make inappropriate demands for healthcare. These inappropriate demands are reconstituted as uncontrolled demand on the very finite time and energy of health professionals in practice, and later, as unsustainable demand in the language of policy and healthcare provision at a societal level. Disciplining demand becomes, then, an important aim for telecare. The rhetoric of such shifts is always an enabling one: users – both patients and professionals – are enabled and empowered, while services are more available and increasingly efficient. The evidence for the former is patchy [15] and, for the latter, it is uncertain [1].

It happens that the ARCHIE framework is useful when we start to consider the political assumptions that underpin technological change, too. The crushing assumption behind much development of telecare is that it is cheaper and more efficient, and that its users are satisfied. The case studies presented by Greenhalgh et al. [3] are ones that might suggest otherwise. They seem to show how telecare distributes more work amongst fewer people, and makes being a patient more difficult because it adds a new burden of co-ordinating and managing technologies and fragmented services. One thing that ARCHIE might usefully do is form a means of ‘designing in’ to sociotechnical systems in healthcare opportunities for minimally disruptive medicine [16] – healthcare interventions that are truly patient-centred – and that reduce the burden of treatment [9,17] on patients and their families.

## Conclusions

User centredness and realism in the design, implementation, and evaluation of new modes of organising and delivering healthcare are important. The ARCHIE framework brings a new set of ethical standards to understanding these. However, it is important, too, to consider the meso-level dynamics that shape these processes. Ideas about demand, choices about services, expectations of patients, and requirements of healthcare workers all form a set of moral and political assumptions that are frequently left outside from debates regarding patient empowerment through new healthcare systems. The ARCHIE Standards give us a new point of departure to consider this problem and to act upon it.
